# Fibroblast growth factor receptor signalling dysregulation and targeting in breast cancer

**DOI:** 10.1098/rsob.210373

**Published:** 2022-02-23

**Authors:** Chiara Francavilla, Ciara S. O'Brien

**Affiliations:** ^1^ Division of Molecular and Cellular Function, School of Biological Science, Faculty of Biology, Medicine and Health (FBMH), University of Manchester, Manchester M13 9PT, UK; ^2^ The Christie Hospital NHS Foundation Trust, Wilmslow Road, Manchester M20 2BX, UK; ^3^ The Manchester Breast Centre, University of Manchester, Wilmslow Road, Manchester M20 4GJ, UK

**Keywords:** breast cancer, fibroblast growth factor receptor, signalling, FGFR inhibitors

## Abstract

Fibroblast Growth Factor Receptor (FGFR) signalling plays a critical role in breast embryonal development, tissue homeostasis, tumorigenesis and metastasis. FGFR, its numerous FGF ligands and signalling partners are often dysregulated in breast cancer progression and are one of the causes of resistance to treatment in breast cancer. Furthermore, FGFR signalling on epithelial cells is affected by signals from the breast microenvironment, therefore increasing the possibility of breast developmental abnormalities or cancer progression. Increasing our understanding of the multi-layered roles of the complex family of FGFRs, their ligands FGFs and their regulatory partners may offer novel treatment strategies for breast cancer patients, as a single agent or rational co-target, which will be explored in depth in this review.

## Graphical abstract

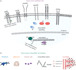

## 1. Introduction

Since the 1980s, there has been an explosion of novel molecular targets to guide drug development strategies for cancer treatment [1]. In breast cancer, this has translated to increased systemic therapeutic options, which, alongside refinements of the diagnostic pathway, have improved median survival (Cancer Research UK 2019, https://www.cancerresearchuk.org/health-professional/cancer-statistics-for-the-uk). Sadly, despite these undoubted advances, breast cancer remains second only to lung cancer as the highest cause of female cancer-related mortality. There is an exigency for novel molecular-targeted therapies (MTTs) to improve patient survival outcomes in early and metastatic breast cancer.

Here, we will first explore how the complex Fibroblast Growth Factor Receptor (FGFR) family, its ligands and cofactors regulate fundamental cellular processes in breast cells and tissue. We will highlight the role of the FGFR axis in normal breast development and in signalling dysregulation in breast tumorigenesis and treatment resistance [2–4]. After discussing the case for anti-FGFR therapeutics as a rational target for drug development in breast cancer, we will outline clinical trial data on the use of FGFR-targeted therapies in cancer patients, to date. Finally, we will focus on future perspectives for FGFR-targeted therapy in breast cancer.

## 2. Overview of FGFR signalling

**Figure 1.**
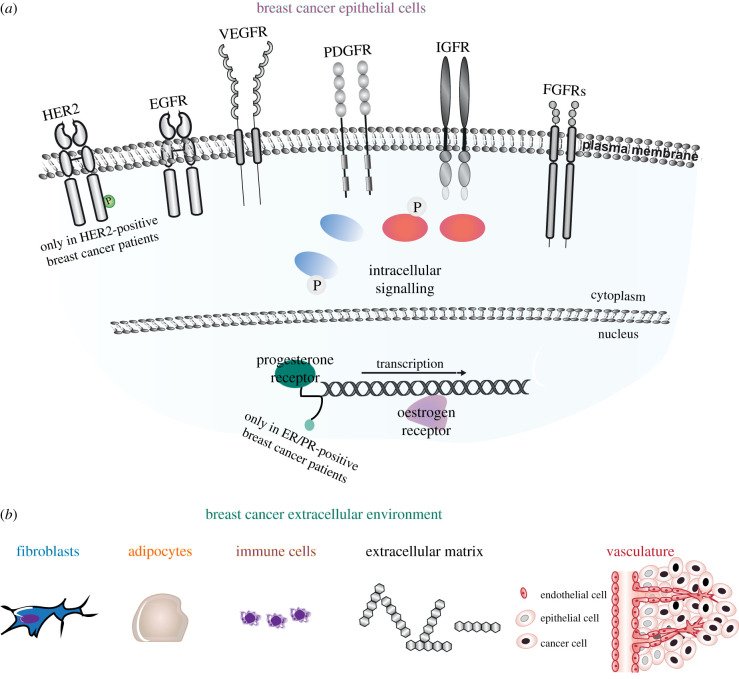
Schematic of the breast cancer epithelial cell and extracellular environment. (*a*) Breast cancer epithelial cells express different combinations of RTKs including EGFR, VEGFR, PDGFR, IGFR and FGFRs, all with a known role in breast cancer. Furthermore, breast cancer epithelial cells express either HER2, or progesterone/oestrogen receptors or none of these s three receptors in HER2-positive, ER/PR-positive and triple negative breast cancers (TNBCs), respectively. (*b*) The breast cancer extracellular environment is composed of fibroblasts, adipocytes, immune cells, and proteins of the extracellular matrix and of the extended vasculature. Cells and proteins are not to scale.

Receptor Tyrosine Kinases (RTKs) are single-pass transmembrane proteins, whose overexpression is associated with breast and other cancers and decreased disease-free survival [5,6]. Among these RTKs, there are epidermal growth factor receptors (EGFRs), vascular endothelial growth factor receptors (VEGFRs), platelet-derived growth factor receptors (PDGFRs), insulin-like growth factor receptors (IGFRs) and FGFRs [6] (figure 1*a*). Upon ligand stimulation, RTKs activate several pathways, including mitogen-activated protein kinase (MAPK), Janus kinase (JAK)/signal transducer and activator of transcription (STAT), phospholipase C gamma (PLCγ) and phosphoinositide 3-kinase (PI3–K) [7] (figure 1*a*). RTK signalling regulates the response of cancer cells to perturbation of the extracellular environment, composed of fibroblasts, adipocytes, immune cells, and proteins of the extracellular matrix and of the extended vasculature [8] (figure 1*b*).

**Figure 2.**
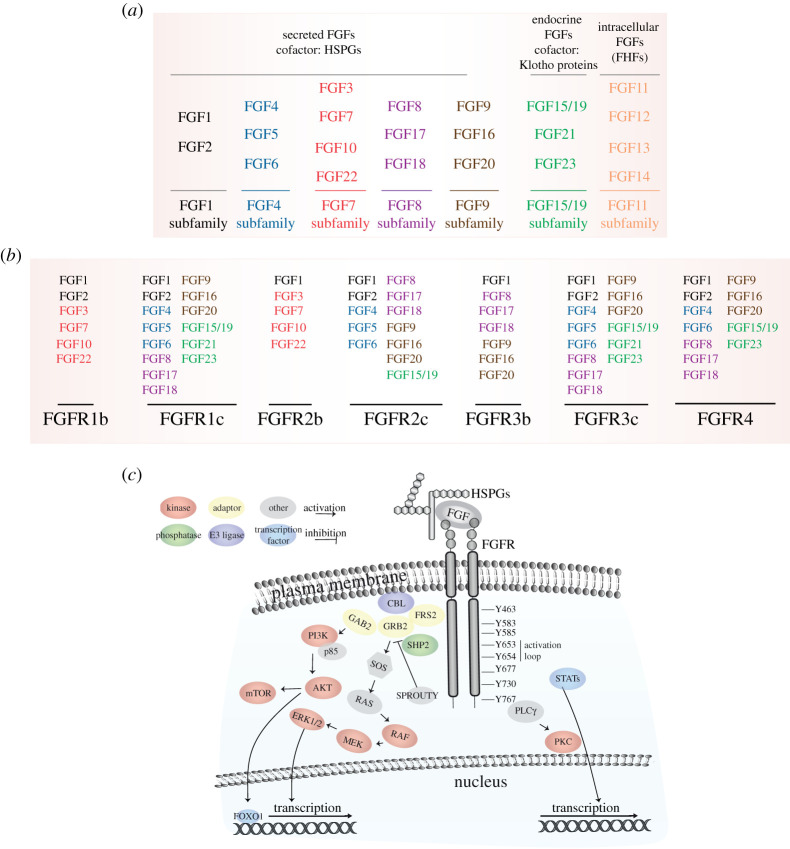
The FGFR signalling players. (*a*) Table showing the FGF subfamilies. (*b*) Table showing the FGF/FGFR pair ligand specificity. FGF colour is based on (*a*). (*c*) Overview of the signalling pathways activated upon FGF binding to FGFR. The numbering in the cytoplasmic domain of FGFR refers to FGFR1c. HSPGs, heparan sulfate proteoglycans.

Here, we will focus on the complex family of FGFRs, their ligands FGFs and their signalling partners. The FGF/FGFR family comprises 18 proteins that bind to and activate four RTKs, FGFRs 1–4. The different subfamilies are based on the biochemical properties, sequence similarities and evolutionary relationships of their members (figure 2*a,b*). Proteins of the extracellular matrix, like Heparan Sulfate Proteoglycans (HSPGs), and cofactors, like the Klotho proteins, regulate the interaction between the FGFs and their receptors at the plasma membrane [4,9,10] (for more details, see §3).

Upon binding of FGF and specific cofactors, dimerization of the FGFR kinase domain induces the phosphorylation of tyrosine (Y) residues leading to full receptor activation and phosphorylation and recruitment of adaptor proteins. Firstly, Y653, in the activation loop, is phosphorylated, followed by the phosphorylation of Y583, Y463, Y654, Y677, Y766 and Y585, whereas Y730 is weakly phosphorylated [11–13] (figure 2*c*). After autophosphorylation, FGFRs are coupled to intracellular signalling pathways, including the RAS-MAPK, PI3K–AKT, PLCγ and STATs pathways [4,14]. FRS2α, which is constitutively associated with the receptor, is phosphorylated upon ligand binding, and recruits adaptor proteins, like GRB2, which in turn recruits SOS followed by members of the MAPK family, and GAB1, which then activates the PI3K–AKT signalling axis [15] (figure 2*c*). PI3K can also be activated directly upon FGF10 binding of FGFR2b [11]. The MAPK pathway, including ERK1/2, JNK and p38 kinases [16–18], regulates transcription [19] and recruits negative signalling regulators like the E3 ligase CBL, the adaptor SPROUTY and the phosphatase SHP2 [20,21]. By contrast, AKT induces the activation of the mTOR complex 1 [22] and the phosphorylation of the FOXO1 transcription factor [23] (figure 2*c*). Following recruitment to activated FGFR the enzyme PLCγ induces calcium ion release, resulting in the activation of downstream kinases like PKC [24] (figure 2*c*). Finally, FGFR also activates STAT1, 3 and 5, which regulate gene expression in the nucleus [25] (figure 2*c*).

## 3. FGFs, FGFRs and cofactors

The FGF family includes secreted FGFs—the FGF1, FGF4, FGF7, FGF8, FGF9 and FGF15/19 subfamilies—and intracellular FGFs—the FGF11 subfamily [26,27] (figure 2*a*). Secreted FGFs are expressed ubiquitously and play crucial roles in early embryonic differentiation and development; during organogenesis of the heart, limb, lung, mammary gland, pancreas, liver, kidney, ear and brain; and in the homeostasis of adult tissues, where they are important for tissue maintenance, repair in wound healing, regeneration and metabolism [28–37]. Secreted FGFs can function as autocrine, paracrine or endocrine factors, and regulate all fundamental cellular processes, including proliferation, survival, migration and differentiation [4]. In addition to the canonical FGF functions, endocrine FGFs of the FGF15/19 subfamily also mediate phosphate, bile acid, carbohydrate and lipid metabolism [38,39]. Whereas FGFs require HSPGs to fully activate their receptor, endocrine FGFs have reduced affinity for HSPGs and signal through the Klotho family [38]. The intracellular FGFs—also known as FGF homologous factors (FHFs) (figure 2*a*)—serve as cofactors for members of the voltage-gated sodium channel family, mitogen-activated protein kinase 8-interacting protein 2 (MAPK8IP2), β-tubulin and NF-κB essential modulator (NEMO) [40–43]. They function as essential regulators of neuronal and myocardial excitability, but their role during embryonic development and human diseases is less clear [44].

The FGF receptors (FGFRs) contain about 800 amino acids in several domains, including three extracellular immunoglobulin-like domains (I, II and III), a transmembrane domain and two intracellular tyrosine kinase domains. There are five *FGFR* genes, and *FGFR1–3* can be alternatively spliced into variants of immunoglobulin-like domain III, referred to as ‘b’ and ‘c’, which are essential for ligand-binding specificity and are mainly expressed in epithelial and mesenchymal cells, respectively [4,45–47] (figure 2*b*). FGFRL1/FGFR5, which is a membrane protein of about 500 amino acids with three extracellular immunoglobulin-like domains (I, II and III), a transmembrane domain and a short intracellular tail with no tyrosine kinase domain [48], will be not considered further. All FGFR isoforms can be activated upon FGF1 binding, although FGF1 has different affinities for each FGFR isoform [26,27]. FGFR1c and 3c interact with FGF2, members of the FGF4, 8 and 9 subfamilies, and with the endocrine FGFs (figure 2*b*). FGFR2c can bind the same FGFs as the other two mesenchymal isoforms of FGFR with the exception of the endocrine FGFs FGF21 and FGF23 [4] (figure 2*b*). The epithelial isoforms of FGFR1 and 2, FGFR1b and 2b, are preferentially activated by the FGF7 subfamily—with FGF7 specific for FGFR2b, whereas FGFR3b interacts with the FGF8 and 9 subfamilies [49,50] (figure 2*b*). The endocrine FGF15/19 subfamily has higher affinity for the mesenchymal isoforms of FGFR1–3 [4] (figure 2*b*). Finally, FGFR4 is activated not only upon FGF2, 4, 6, 8, 17 and 18 binding but also by the endocrine ligands FGF15/19 and FGF23 [51] (figure 2*b*). It is not known whether each FGFR isoform regulates specific downstream signalling outputs, although some evidence exists that FGFR1 signalling is similar to FGFR2 signalling but differs from FGFR3-4 signalling [4]. However, this variety of FGF/FGFR pairs influences cellular signalling architecture and downstream responses in all cells and tissue. For instance, mesenchymal tissues expressing FGFR1-2c are often activated by FGF ligands that are expressed in the surrounding epithelial cells, such as members of the FGF4 and FGF8 subfamilies, whereas epithelial tissues express FGFR1-2b and bind ligands secreted from mesenchymal tissues (e.g. FGF7 subfamily) [4] (figures 1 and 2*b*). This paracrine expression of ligands and receptors is crucial during FGFR-mediated development of branching organs, like the mammary gland [31], and has been found dysregulated in human cancers [2,10].

HSPGs, α-Klotho and β-Klotho are potent cofactors for FGFR signalling activation [52,53]. Heparan sulfate consists of chains of repeating sulfated disaccharides linked to *N*-acetylglucosamine; these chains are covalently linked to syndecan, perlecan and other transmembrane or cell surface-anchored core proteins, or diffusible proteins of the extracellular matrix [54]. HSPGs enhance the activity of FGFs by regulating the binding, stability, specificity and affinity of FGF/FGFR pairs, and sequester the FGFs by limiting the diffusion of secreted FGFs through the extracellular matrix [53]. This is crucial during development, as differences in binding affinity of FGF7 and FGF10 for HSPGs generate a gradient of FGFs whichthat regulate epithelial branching during organogenesis [55,56]. The endocrine FGFs with reduced affinity for HSPGs require the single-pass transmembrane proteins α-Klotho and β-Klotho for receptor binding [52]. α-Klotho, which is expressed in the kidney and in the brain, was identified as a cofactor for FGF23 signalling through FGFR1c, FGFR3c and FGFR4 to regulate phosphate and calcium homeostasis [57]. β-Klotho is mainly expressed in the liver and white adipose tissue and is required by FGF15/19 and FGF21 to activate FGFR4 and FGFR1c, respectively [58]. The Klotho cofactors can also directly compete with the FGF8 subfamily members for the binding of their receptors, thus inhibiting their actions while activating endocrine FGFs [59].

Genetic alterations of the *FGF*, *FGFR* or *cofactor* genes—like mutations, Single Nucleotide Polymorphisms (SNPs) or amplifications—have been shown to affect cellular responses during development, in genetic diseases and cancer, including breast cancer [3,60,61]. This is demonstrated by the variety of phenotypes observed in *Fgf, Fgfr, Hspg* and *Klotho* knock-out mice (summarized in [4]), reflecting the fundamental roles that FGFR signalling has in several pathological conditions. For instance, mice not expressing *Fgfr2b* or its ligand *Fgf10* present impaired signalling underlying the formation of branching organs, including lungs, kidney or mammary gland (see §4.2) and die at birth (P0). Therefore, FGFRs, FGFs, signalling cofactors and partners require robust and specific regulatory mechanisms to guarantee the activation of the right output in the right condition in both physiological and pathological conditions.

## 4. FGFR signalling in health and disease

### 4.1. FGFR signalling regulation in tissue homeostasis

**Figure 3.**
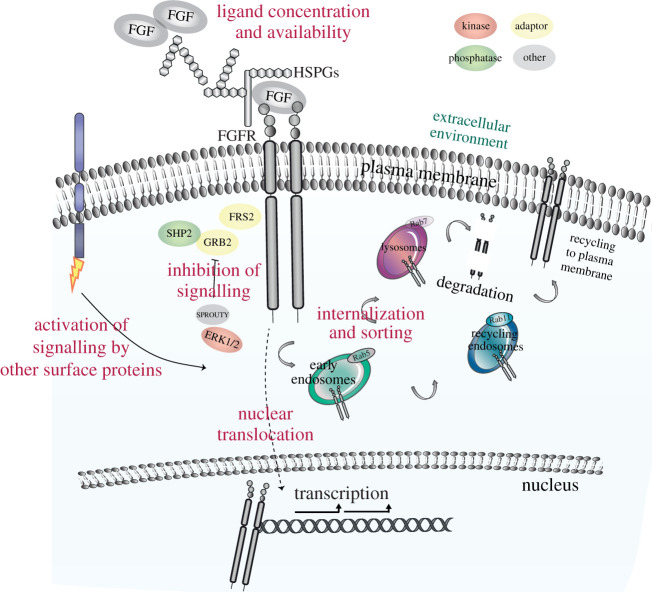
Mechanisms of regulation of FGFR signalling. Schematic of the main regulatory mechanisms of FGFR signalling, including ligand concentration and availability in the extracellular environment, the presence of co-partners on the plasma membrane, signalling inhibitors and signal localization.

Aberrant activation of FGFR signalling in cells and organs is associated with development defects, genetic and metabolic disorders, and cancer. Therefore, FGFR signalling needs to be tightly regulated. Regulatory mechanisms may occur at different levels: in the extracellular environment, at the plasma membrane, during internalization and sorting or translocation to the nucleus, and through feedback signalling mechanisms (figure 3). The presence and the amount of different FGFs in the extracellular matrix affect downstream signalling architecture. For instance, FGF7 and FGF10, which activate FGFR2b [62], induce transient and sustained ERK1/2 phosphorylation in epithelial cells, which results in opposite long-term outputs, proliferation and migration, respectively [11]. The concentration of these two members of the FGF7 subfamily in the extracellular environment triggers also specific branching patterns during organogenesis [56]. Thus, the different affinity of FGF7 and FGF10 for HSPGs is a key regulatory mechanism underlying the response to these FGFs in epithelial cells [55]. Other cell surface molecules, like anosmin-1, Similar Expression to Fgf (SEF), and the adhesion molecules *N*-cadherin or Neuronal Cell Adhesion Molecule (NCAM), can modulate FGFR signalling in different cell contexts and organs [63–67], thus enlarging the variety of extracellular modulator of FGFR signalling [10] (figure 3). We have recently shown that EGFR regulates FGFR2b trafficking and signalling outputs in response to FGF10 stimulation [68]. In addition to FGFRs, FGFs and the cofactors HSPGs or Klotho proteins, the transcript and protein levels of these cell surface molecules change during development and in pathological conditions, thus fine-tuning FGFR signalling activation [10].

Differences in FGF-induced dimerization of FGFRs on the cell surface result in specific phosphorylation patterns on the receptor and in the recruitment of distinct signalling partners, thus determining the amplitude and dynamics of cellular responses [69]. This would explain how the formation of a stable FGF10/FGFR2b complex results in the phosphorylation of Y734 on FGFR2b, activation of ERK1/2 in a sustained manner, and regulation of cell migration [11]. By contrast, FGF7, which has less affinity for FGFR2b and for HSPGs compared with FGF10 [26,27,55], is not able to induce Y734 phosphorylation, thus leading to transient ERK1/2 phosphorylation and cell proliferation upon FGFR2b binding [11,69]. Similarities and differences in signalling of the four FGFRs could be also determined by sub-cellular localization (figure 3). For instance, the four FGFRs follow a different route after FGF1-induced internalization, as FGFR4 is recycled to the cell surface, whereas FGFR1–3 are degraded into lysosomes [70]. FGFR2b has been shown to be sorted into the degradative route upon FGF7 binding, and into recycling endosomes in response to FGF10 [11,71]. This different FGFR trafficking results in the activation of specific downstream signalling pathways [11,63,72]. Furthermore, nuclear translocation of FGFR1 and FGFR2 has been shown to specify the behaviour of cancer cells by regulating signalling and transcription [73,74], thus adding a further layer of complexity in the regulation of FGFR activity (figure 3).

Inhibitory mechanisms of signalling are also important for the precise regulation of FGFR functions in physiological and pathological conditions. For instance, GRB2 direct interaction with FGFR2c does not enable the recruitment of signalling adaptors to the C-terminus of the receptor, thus resulting in signalling attenuation [75]. The MAPK pathway can also exert negative feedback inhibition of FGFR signalling by directly phosphorylating the C-terminus of FGFR2 at serine 777 [76]. This is also an example of how other RTKs can regulate the activity of FGFRs by using members of the MAPK family. The ubiquitously expressed family of SPROUTY is a known negative regulator of RTK signalling. SPROUTY interacts with GRB2 to regulate the activation of MAPK and PI3K–AKT downstream from FGFR phosphorylation [77,78] (figure 3). Phosphatases like DUSP6 or SHP2 attenuate the FGFR/MAPK signalling axis during development, and their deregulation may lead to cancer formation [79,80]. Finally, the E3 ubiquitin ligase CBL induces the ubiquitylation of FGFR after receptor internalization and regulates the sorting of FGFR into the degradative pathway which results in signalling termination [81] (figure 3).

The combination of all these regulatory mechanisms plays a crucial role in modulating the nature, specificity, dynamics and amplitude of FGFR signalling outputs during homeostasis.

### 4.2. FGFR signalling in mammary gland development

FGFR signalling plays a crucial role in mammary gland formation during development. Mammary gland formation in the mouse begins around embryonic day 10 (E10) [2,82]. FGFR2b signalling is required for placode induction and development, as shown by the disappearance of mammary placode in *Fgfr2b* knock-out mice due to decreased proliferation of the epithelium [31]. However, in *Fgf10* knock-out mice this defect is less pronounced, owing to the redundant expression of another FGFR2b-specific ligand, FGF7 [31] (figure 2*a,b*). The expression of FGFR2b remains elevated in virgin mice but decreases during pregnancy and lactation. Indeed, the postnatal phase of mammary gland development is characterized by high ramification of the gland, which is mainly due to the FGF10/FGFR2b signalling, with FGF7 playing a minor role [31].

A crosstalk between FGF10 and WNT signalling has been described during mammary placode development [31,83], where FGF10 controls the expression of the *Wnt* ligands, including *Wnt10b* [84]. It has been suggested that FGF10 and WNT signalling are both required for regulating cell migration during mammary placode induction [84]. FGF10/FGFR2b signalling is also important for branching of the mammary epithelial tree, which starts at E15.5 but occurs mostly postnatally [85]. Indeed, the mammary gland epithelium fails to ramify in the *Fgf10* knock-out mice, where an underdeveloped mammary fat pad has also been observed [31], suggesting a role for FGF10 in the formation of adipocytes via FGFR1b signalling [86] (figure 2*a,b*). During the 2 month period following birth, the mouse mammary gland is subjected to extensive branching through cell proliferation and differentiation events of the terminal end buds (TEBs) of the mammary ducts [2]. The formation of TEBs depends on ER signalling, which probably controls FGFR2b signalling [2]. The TEBs contain mammary progenitors giving rise to both luminal epithelial and myoepithelial cells, and the continuous interaction of TEBs with the surrounding microenvironment is crucial for the maintenance of the lumen structure and for milk secretion [2]. This interaction is lost during early-stage breast cancer and is controlled by paracrine signalling including FGF10/FGFR2b signalling and FGF20 signalling [85,87].

Interestingly, whereas deletion of the *Fgfr1* gene in the mammary epithelium has minor effect on the development of the mammary gland, the simultaneous depletion of *Fgfr1* and *Fgfr2* genes leads to a significant loss of stem cell progenitors and defective epithelial branching [88]. Indeed, the several FGFs produced by the mammary gland stroma, including FGF2, FGF7, FGF9 and FGF10, regulate epithelial morphogenesis through FGFR1 and FGFR2 activation [88] (figure 2*a,b*). The role of these FGFs in the stroma has been recently elucidated [89]. FGF2 and FGF9 regulate sustained signalling activation of primary fibroblasts from mammary gland, and FGF2 could induce fibroblast proliferation, migration and remodelling of the extracellular environment [89]. Altogether, these data define a crucial role for FGFR signalling in the development of mammary gland both pre- and postnatally and suggest a role also in remodelling of the extracellular environment and in the formation of early lesions during breast cancer.

### 4.3. FGFR signalling in breast cancer

Dysregulation of FGFRs, FGFs and downstream signalling molecules has been described in breast cancer [2,89]. The term breast cancer encompasses a heterogeneous group of invasive cancers, whose cell of origin derives from primary breast tissue and where biological aggressiveness is indicated by tumour grade and proliferation rate [90]. Commonly, breast cancers will arise within cells (or their developmental progenitors) lining the ducts or composing the lobules of the glandular breast. This ductal/ lobular cellular bilayer is encapsulated by the diverse cell types (immune and non-immune) of the tumour microenvironment (TME) and stromal tissue (figure 1*b*). Breast cancer may be defined into three broad clinical subtypes ((hormone receptor (ER/PR)-positive (approx. 70%), HER2-positive (approx. 20%) and triple negative breast cancer (TNBC, approx. 10%)), using laboratory-based immunohistochemistry and/or *in situ* hybridization assays based on differential expression of oestrogen, progesterone and the HER2 receptor (ER, PR and HER2) (figure 1*a*). To date, these broad clinical subtypes have been the tenets of therapeutic decision-making. On a genomic level, at least four intrinsic molecular breast cancer subtypes can be defined in breast cancer tissue, including luminal A, luminal B, HER2-positive and basal-like breast cancers [91]. Intrinsic molecular subtype, as defined, for example, by PAM-50 classification [92], may be used as a risk predictor for adverse clinical outcomes [93]. Current clinical trial strategies may combine clinical and molecular subtypes to guide treatment personalization and validate clinical decision-making (e.g. [94,95]), and/or pre-select (pre-screen) patient population for predictive biomarkers indicative of treatment sensitivity [96].

**Figure 4.**
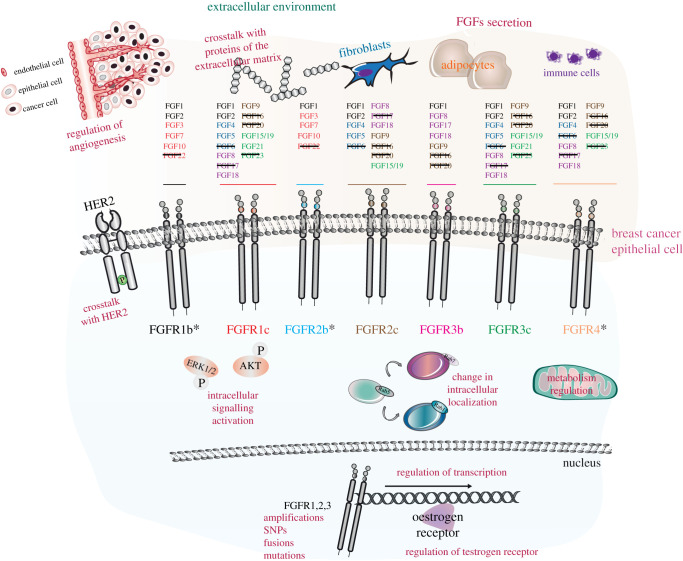
Roles of the FGFR family in breast cancer. Schematic of breast cancer extracellular environment (from figure 1) and breast cancer epithelial cells (from figures 1 and 2). The asterisks indicate the three main FGFR isoforms with a critical role in breast cancer progression. The text in burgundy indicates the mechanisms underlying FGFR roles in breast cancer, including secretion of FGFs, interactions with proteins of the extracellular matrix, regulation of vasculature during angiogenesis, crosstalk with HER2, regulation of signalling, metabolism and transcription, changes in intracellular localization, and genomic alterations, as described in the main text. The black lines on the ‘FGF’ text indicate the lack of available information on the role of those specific FGFs in breast cancer.

The bioinformatic analysis of available data from BioPortal (http://www.bioportal.no/) suggests that *FGFR1* gene alterations are as significant in breast cancer patients as alterations in other known drivers of breast cancer (e.g. HER2) [97]. Furthermore, several studies have shown that FGFR signalling is important for the growth of breast cancer cells *in vitro* [98–102]. Activation of FGFRs in breast cancer is attributed to receptor gene amplification and mutations, gene fusions resulting from translocations and amplification, and more rarely alternative splicing [3] (figure 4). Mutations cause constitutive activation of FGFR signalling, whereas changes in FGFR isoforms increase the FGF/FGFR pairs capable of inducing cell growth [3]. The first studies on the role of FGFR in breast cancer identified the amplification of *FGFR1* and *FGFR2* genes in human breast cancer samples [103]. Since then, genome-wide association studies [104] have associated SNPs within the *FGFR2* gene with increased breast cancer susceptibility [105]. Very recently, genetic alterations of *FGFR*s have been associated with breast cancer metastases and included in the list of actionable targets in breast cancer [106–108]. In metastatic breast cancers, *FGFR*s are for instance implicated in poor response to chemotherapy [109] and resistance to targeted therapies [110]. FGFR dysregulation may also play a role in organ-specific metastases in breast cancer. In a recent study, FGFR1 and p53 mutation was associated with central nervous system (CNS) metastases in a breast cancer patient cohort [108] and *FGFR2* amplification was reported as a clonal event in CNS metastases in a warm autopsy series [111]. FGFR4-induced genomic signature was also found to be predictive of organ-specific metastases (brain, liver, lung) in the Molecular Taxonomy of Breast Cancer International Consortium (METABRIC) breast cancer patient cohort independent of clinical subtype or stage [112].

Numerous *FGF* genes are amplified or show deregulated levels of transcripts and proteins and are often overexpressed in the extracellular matrix or in the stroma, all mechanisms contributing to the amplification of FGFR signalling or the inhibition of its regulatory mechanisms [113] (figure 4). Furthermore, FGFs can act synergistically with VEGF to amplify tumour angiogenesis [114]. The tumour microenvironment consists of cancer cells, adipocytes and stromal/immune cells, such as fibroblasts, endothelial cells, lymphocytes and macrophages, and is involved in cancer cell proliferation, invasion and metastasis [115] (figures 1 and 4). FGFs deriving from cancer cells or stromal cells and their receptors on cells of the breast tumour microenvironment are key players for the regulation of tumour cell remodelling [116,117], immune surveillance and evasion [118], and response to therapies [119]. Resistance to anti-cancer therapies has been attributed not only to overexpression of FGFs, but also to increased abundance of FGFR and members of the FGF/FGFR signalling axis, like MAPK [120,121].

We present below an overview of the known roles of the FGF subfamilies and of FGFR1–4 in breast cancer progression and metastasis. We will highlight their known genetic alterations, and discuss how dysregulated signalling pathways and cellular localization, and interactions with the microenvironment globally affect the behaviour of breast cancer cells (figure 4).

## 5. FGFs in breast cancer

### 5.1. The canonical FGFs

#### 5.1.1. The FGF1 subfamily: FGF1, FGF2

The FGF1 subfamily is composed of FGF1 (also known as acidic FGF) and of FGF2 (also known as basic FGF) (figure 2*a,b*), which have minor roles during development, but are crucial for tissue repair after injury in the adult and in angiogenesis [4]. Both FGF1 and 2 are present in the breast, with FGF2 localized to myoepithelial cells of normal breast, and signal through FGFR1, 2 and 4 [122] and HSPGs [123]. Very recent data suggest that the stabilization of FGF2 changes the nature and the dynamics of FGFR signalling in primary mammary fibroblasts, suggesting a crucial role for the FGF2 interaction with extracellular matrix proteins, like HSPGs, in dictating breast cancer signalling [124]. FGF2 regulates tumour growth and migration *in vitro* and in xenograft models [125], not only by activating FGFR1 signalling [126], but also through oestrogen receptor (ER) signalling [127] (figure 4). The overexpression of its high molecular weight isoform induces lung metastasis and confers endocrine resistance in pre-clinical models [128]. Both FGF1 and FGF2 play a crucial role in breast cancer angiogenesis [129,130] (figure 4). It would be interesting to block either FGF or both using available single-chain variable fragment (scFv) antibodies and their dimerization form, which have been shown to inhibit FGF1-dependent breast cancer growth *in vitro* [131,132]. This strategy is based on the idea of inhibiting FGF signalling with ligand-trap molecules or antibodies and not the receptor or downstream signalling players [133].

#### 5.1.2. The FGF4 subfamily: FGF4, FGF5, FGF6

The FGF4 subfamily is composed of FGF4, which is crucial for early development in mice, FGF5 and FGF6, whose deletion in mice does not have a known phenotype [4] (figure 2*a,b*). In the context of breast cancer, the expression of *FGF5* and *FGF6* was detected at very low level in comparison with *FGF1* and *FGF2* [134]. By contrast, the *FGF4* gene is amplified in breast cancer together with *FGF3* and *FGF15/19* as they are all part of the locus on human chromosome 11q13 that is frequently amplified in several tumours [135]. To the best of our knowledge, FGF6 signalling has not been associated with any phenotype in breast cancer neither *in vitro* nor *in vivo*, whereas FGF4 and FGF5 seem to have overlapping but also specific roles. Both FGF4 and FGF5 regulate resistance to HER2 inhibitors. FGF4 promotes resistance to lapatinib in HER2-positive breast cancer cell lines through FGFR1 signalling [136], and FGF5 by inducing FGFR2 activation, which in turns transactivates HER2 and promotes resistance [137]. It would be worth investigating whether FGF4 and FGF5 act synergistically to promote resistance mechanisms or whether their role depends on other clinical parameters. FGF4 signalling regulates breast cancer cell migration and invasion [138–140], whereas FGF5 seems to have a specific role in the formation of bone metastasis as shown by its overexpression in metastatic samples compared with normal breast [141]. However, it has also been reported that low expression of FGF5 correlates with a protective role in breast cancer patients [142]. These contrasting results need further studies to uncover the mechanisms underlying FGF5 signalling in breast cancer.

#### 5.1.3. The FGF7 subfamily: FGF3, FGF7, FGF10, FGF22

Members of the FGF7 subfamily are FGF3, FGF7, FGF10 and FGF22 (figure 2*a,b*), of which FGF10 is known to be involved in the formation of mammary gland during development [2] (see §4.2). The *FGF3* gene is amplified in breast cancer [135] and this correlates with a lower response in patients with HER2-positive breast cancer treated with anti-HER2 therapy [136]. FGF3 levels correlate with stage and grade, FGFR2 signalling activation and proliferation of breast cancer cells [143]. Therefore, anti-FGF3/FGFR2b therapies may benefit patients with HER2-positive breast cancer (figure 4). FGF7 and FGF10 are among the components of breast cancer organoid growth medium [144], indicating that they both play a crucial role in the initiation and/or maintenance of breast cancer. FGF7 is detected in both stroma and tumour cells [145] and increases breast cancer cell proliferation and migration *in vitro* [146–148]. Interestingly, AKT signalling is required for such FGF7-mediated regulation of tumour progression [148,149]. In terms of signalling pathway activation, FGF7 binding to its receptor FGFR2b (figure 2*b*) also activates ERK1/2, which in turn promotes FGF7-dependent migration of ER-positive breast cancer cells [150]. Furthermore, FGF7/FGFR2 signalling induces the downregulation of progesterone receptor (PR) via the kinase RSK2, which correlates with poor prognosis in the clinic [151]. Finally, the FGF7/FGFR2 signalling axis increases ER phosphorylation, ubiquitination and subsequent ER proteasomal degradation, which results in resistance to tamoxifen treatment [152]. Altogether, these data show that FGF7 signalling through FGFR2b is crucial for promoting breast cancer through different mechanisms and drives resistance to conventional therapies.

FGF10 is also expressed exclusively by the stromal fibroblasts of normal and breast cancer tissue and has been reported to be an oncogene in mammary tumour virus mouse models and in a subset of breast carcinomas showing high expression of the protein [153,154] (figure 4). FGF10 expression increases if the rs10941679 SNP is present, which in turn would increase risk of breast cancer in patients expressing the *FGFR2* SNP rs2981578 variant [155,156]. This is due to the paracrine action of FGF10 on its receptor, the FGFR2b isoform, which is highly abundant on the mammary gland [157] (figure 2*a,b*). FGF10 regulates Epithelial to Mesenchymal Transition (EMT), cell viability, migration and colony formation in breast cancer cell lines by increasing the expression of mesenchymal factors (such as vimentin, *N*-cadherin, snail, slug, TGF-β), and ERK1/2 and PI3K–AKT signalling [2,158]. FGF10 stimulation of the ER-positive breast cancer cell line MCF-7 decreases dependency on oestrogen and sensitivity to treatment with anti-oestrogen [159], suggesting that anti-FGF10/FGFR inhibitors can be used to bypass resistance to anti-hormone therapies.

In conclusion, the FGF7 family—except for FGF22 whose role in breast cancer has not been reported yet—plays a crucial role not only in the formation of mammary gland during development, but also in breast cancer initiation, progression and resistance to therapies.

#### 5.1.4. The FGF8 subfamily: FGF8, FGF17, FGF18

The FGF8 subfamily is composed of FGF8, FGF17 and FGF18 (figure 2*a,b*). FGF8 is crucial for early- stage development, FGF17 controls development of the brain, and FGF18 is essential for multiorgan development as *Fgf18* knock-out mice die at birth similar to the *Fgf10* knock-out mice [4]. FGF8 expression is higher in malignant breast tissue compared with normal cells [160]. As the canonical FGF8 receptors FGFR2c and FGFR3c are expressed at low level in breast cancer cells, it is possible that FGF8 acts in an autocrine manner on FGFR1 and FGFR4, which are instead present in the breast epithelium [160] (figures 2*b* and 4). It has been reported that one of the FGF8 isoforms, FGF8b, increases anchorage-independent growth *in vitro* and vascularization in nude mice [161,162], suggesting that FGF8 is involved in the induction of transformation and in angiogenesis in breast cancer. Given the role of FGF8b in regulating the differentiation of osteoblasts, a potential role of FGF8b may include driving the formation of osteosclerotic bone metastases [163]. FGF8 signalling increases oestrogen-induced breast cancer cell proliferation by inducing the expression of the ER mRNA, and at the same time suppresses the inhibition of mitosis by activating the cell cycle regulator CDC2 and other regulators of cell cycle entry [164,165]. The increased expression of FGF18 mRNA and protein has been associated with migration *in vitro* and poor overall survival in cancer patients [166]. A recent study showed that FGF18 increased cell migration and EMT through AKT signalling and by inducing the transcription of proliferation-related genes, including *CDC2*, metastasis-related genes (*TGFβ*, *MMP-2* and *MMP-9*) and EMT markers like the SNAIL proteins and *N*-cadherin [167]. Both FGF8 and FGF18 have a role in regulating the cell cycle of breast cancer cells, a finding that deserves further investigation in the search for novel potential treatments for patients with highly proliferative breast cancer.

#### 5.1.5. The FGF9 subfamily: FGF9, FGF16, FGF20

Members of the FGF9 subfamily (FGF9, FGF16, FGF20 (figure 2*a,b*)) regulate development at multiple levels in lungs, male germ cells, skeleton, small intestine and cardiomyocytes [4]. FGF16 and FGF20 do not have a known role in breast cancer progression. On the contrary, FGF9 is highly expressed in breast cancer compared with normal tissue, although its expression is not as high as the expression of other FGFs like FGF1 [134] (figure 4). FGF9 is capable of inducing cancer stem-like cell properties in breast cancer cell lines and freshly isolated breast cancer cells through FGFR activation [168]. Furthermore, a recent publication suggested a role for FGF9 in resistance to the commonly used anti-cancer agent gemcitabine [169].

### 5.2. The endocrine FGFs: FGF15/19, FGF21, FGF23

The endocrine FGFs have several roles both during development and in the homeostasis of adult tissues [4] (figure 2*a,b*). *Fgf15* knock-out mice present defects in cardiac development and deficiency in the intestine functions through cell cycle regulation; *Fgf21* depletion does not affect development but profoundly impairs metabolism in fasting conditions; and *Fgf23* knock-out mice die at birth owing to increased levels of vitamin D, hyperphosphataemia and cardiac defects [4]. The human *FGF19* gene is amplified in breast cancer together with *FGF3* and *FGF4* [135], and this correlates with worse prognosis in invasive ductal breast carcinomas, particularly in older patients with lymph node metastasis and negative ER status [170]. Genetic knock-out of *FGF19* decreases breast tumour progression and metastasis in either mouse models of breast cancer or experimental metastasis models [171]. The authors of this discovery demonstrated that FGF19 activates the AKT signalling pathway through FGFR4, and that this is critical for the oncogenic role of FGF19 [171]. Given the role of FGF21 in metabolism and the important relationship between metabolism and breast cancer [172], it has been suggested that monitoring the serum levels of FGF21 during anti-breast cancer therapies could be valuable, although more data are necessary to shed light on the role of FGF21 in breast cancer [173] (figure 4).

### 5.3. The intracellular FGFs: FGF11, FGF12, FGF13, FGF14

Mice knocked-out for intracellular FGFs (figure 2*a,b*) present various defects in neurons (*Fgf13* and *Fgf14* knock-out) and altered voltage-gated sodium channel physiology (*Fgf14* knock-out) [4]. FGF13 and FGF14 might be tumour suppressors in breast cancer. Indeed, a long non-coding RNA, FGF13-AS1, inhibits breast cancer cell proliferation, migration and invasion by reducing the half-life of Myc, and of insulin-like growth factor 2 mRNA binding proteins (IGF2BPs) [174]. On the same lines, the expression of a long non-coding RNA, FGF14 antisense RNA 2, was downregulated in breast compared with normal tissue, and this correlates with larger tumour size and more lymph node metastasis [175] (figure 4). By contrast, other studies show that FGF13 may promote metastasis by altering breast cancer cell migration, especially in TNBC [176,177]. Further analyses of patient-derived samples and experiments are needed to clarify these results.

## 6. FGFRs in breast cancer

### 6.1. FGFR1

The *FGFR1* gene on the 8p11–12 chromosomal region is mutated in around 15% of breast cancer, more specifically in 27% of HER2-positive patients, in 23% of ER-positive patients and in 7% of TNBC patients [3,104,178]. This chromosomal region is amplified simultaneously with the 11q12–14 region, which contains other oncogenes with a role in breast cancer progression, like *CCND1*, *FGF3*, *FGF4* and *FGF19* [135]. However, the fact that translocations and mutations of the *FGFR1* gene lead to a constitutively activated FGFR1 protein in around 10% of tumours [179–181] highlights the unique role of FGFR1 as an oncogene and its accountability during the arise of resistance. Indeed, besides correlating with low survival rates in lobular and metastatic breast cancer [109,182], *FGFR1* gene amplification is implied in resistance to hormone therapies [183], to anti-HER2 treatments [136] and to CDK4/6 inhibitors [184]. *In vivo*, resistance to CDK4/6 and to anti-HER2 treatment inhibitors can be reversed by combination with anti-FGFR drugs, such as lucitanib or erdafitinib [136,184]; this indicates the importance of targeting different pathways in breast cancer (figure 4). *FGFR1* gene fusions, which account only for 8% of the total gene aberrations, have been observed with *Fop*, *Bcr* and *Znf198* [104]. Despite the rarity of kinase fusion events in breast cancer, these events showed an enrichment in hormone-resistant samples and in metastasis [185].

FGFR1 signalling has been shown to be amplified by activating mutations like K656E and N546 *in vitro* and in malignant breast cancer compared with normal breast [13,186]. In the *FGFR1*-amplified cell line MDA-MB-134, treatment with an anti-FGFR1 antibody reduced the phosphorylation of FRS2 and ERK1/2 downstream of the receptor, which resulted in the reduction of tumour growth in pre-clinical models [179]. High levels of FGFR1 are known to induce MAPK activation and subsequent expression of cyclin D, leading to increased cell cycle progression and cell growth [183]. These examples illustrate the transforming ability of FGFR1 signalling in breast cancer cells and point to the FGFR1/MAPK signalling axis as a prominent drug target. Besides regulating cell proliferation, the FGFR1-dependent activation of ERK1/2 is implicated in epithelial to mesenchymal transition (EMT) by stabilizing the transcription factor Twist in HER2-positive breast cancers [187]. This may represent one of the mechanisms responsible for acquired resistance to the anti-HER2 drug lapatinib [187], and this finding suggests that inhibiting both FGFR1 and HER2 signalling might be beneficial for patients (figure 4).

FGFR1 regulates EMT also through integrin β3 signalling, which disrupts the known interaction between FGFR1 and E-cadherin on epithelial cells and leads to redistribution of FGFR1 in sub-cellular compartments [188]. This cellular mechanism favours three-dimensional outgrowth of metastatic breast cancer cells in the presence of FGF2 *in vitro* and correlates with decreased survival in patients with basal-like breast cancer [188]. On the contrary, the AKT–mTOR signalling pathways are involved in FGFR1-dependent regulation of anoikis and autophagy, thus contributing to the tumorigenic activity of FGFR1 [189]. In addition to AKT and ERK1/2 activation, FGFR1 induces the activation of the IGF1R pathway through the recruitment of IRS1 in breast cancer cells resistant to the inhibitor metformin, thus suggesting a connection between growth factor mitogenic signalling and glucose metabolism in breast cancer cells [190] (figures 1 and 4). FGFR1 signalling associated with ER in the nuclei of breast cancer cells regulates the transcription of ER-dependent genes, contributing to mechanisms of resistance in ER-positive samples [191] (figure 4). Breast cancer cell behaviour can also be regulated *in vitro* by nuclear translocation of FGFR1b upon FGF10 binding, and this change in sub-cellular localization of FGFR1b regulates transcription and correlates with breast cancer invasion in clinical material and a three-dimensional model of breast cancer [73]. Therefore, the fine regulation of FGFR1 localization-dependent signalling may be a crucial factor contributing to the aggressiveness of breast cancer.

FGFR1 signalling may promote perturbations of the breast cancer microenvironment and immune response which might lead to the formation of metastasis. For instance, FGFR1 is implicated in macrophage-dependent cell migration and invasion by activating the TGFβ/SMAD signalling axis and the receptor for inflammatory cytokines Cxcr2 [192]. These results indicate that macrophages may be important for promoting FGFR1-driven breast cancer metastasis. FGFs activating FGFR1 have the capacity to increase the differentiation of osteoclast; therefore it has been suggested that this would increase FGFR1-dependent migration of breast cancer cells towards the bones, one of the known metastatic sites in breast cancer patients [193]. More recent data have shown that the role of FGFR1 in distant metastasis is to amplify the effect of HER2 overexpression, and this would correlate with a less favourable prognosis in patients co-amplifying both *FGFR1* and *HER2* (8%) compared with patients with either *FGFR1* or *HER2* amplification or without amplification [194]. This idea was confirmed by data showing that FGFR1 amplification was strongly associated with increased risk for distant disease in axillary node-, HR- and HER2-positive early breast cancer [195]. It is interesting to note that there is a difference in the capacity of FGFR1 isoforms to promote an aggressive phenotype, with the FGFR1β isoform inducing higher motility than FGFR1α [196]. The crosstalk between FGFR1 and other signalling pathways in responding to changes of the environment is also exemplified by data showing that the inhibition of both FGFR1 and VEGFR is more efficient in reducing tumour angiogenesis than either treatment alone [197,198] (figure 4).

### 6.2. FGFR2

The *FGFR2* gene is located on the 10q26.13 chromosomal region, which is amplified in only 5% of all breast cancer patients, in particular TNBC patients [199]. In TNBC, *FGFR2* amplification has been associated with robust activation of signalling, cellular transformation and resistance to FGFR inhibitors in pre-clinical models [3,22,103,200]. However, very recently it has been reported that FGFR2 can be expressed also in ER/PR-positive tumours where, surprisingly, low FGFR2 expression correlates with poor prognosis [201] (figure 4). There are 12 known mutations in the *FGFR2* gene reported in the COSMIC database (https://cancer.sanger.ac.uk/cosmic), among which four missense mutations are capable of constitutively activating FGFR2 (N549K, S253R, K660N and P253R) [104,202] and two (M538I and N550 K) contribute to FGFR2-dependent resistance to CDK4/6 inhibitors in ER-positive breast cancers [110,184]. Thus, the position of these mutations on the protein affects the function of FGFR2 in breast cancer, suggesting that multiple regulatory mechanisms for FGFR2 signalling are present *in vivo*. Genome-Wide-Association-Studies (GWAS) have shown that SNPs in the second intron of the *FGFR2* gene are significantly associated with high risk of breast cancer, in particular in post-menopausal women [105,203–205], confirming an oncogenic role for *FGFR2. FGFR2* maintains a population of tumour-initiating cells in mice, and claims have been made that FGFR2 can be targeted to eliminate breast cancer stem cells [206]. From a mechanistic point of view, it has been suggested that histone acetylation modulates access to selected polymorphic sites within intron 2, thus regulating downstream splicing sites, which generates FGFR2c isoforms [207]. As there is also evidence for gene polymorphism of the FGFR2b ligand FGF10 [208], it would be worth exploring in detail the consequences of these nuclear events on the expression of different receptor isoforms and on signalling activation and specificity in both pre-clinical models and patient-derived samples. Interestingly, BRCA-1- and ER-double-positive breast cancers showed not only increased expression of FGFR2 [209] but also the presence of the SNP rs2981582 [210], which is associated with high risk of breast cancer [211]. Therefore, these data suggest that different *FGFR2* SNPs or their combination may contribute to initiation, signalling or elevated risk of breast cancer, highlighting the importance of the analysis of *FGFR2* SNPs as clinical markers or predictors.

Given the complex regulation of the *FGFR2* gene it is not surprising that FGFR2-dependent signalling is deregulated in breast cancer [211]. For instance, the amplification of *FGFR2* results in the activation of PI3K–AKT signalling and inhibition of apoptosis in breast cancer cell lines [199]. Furthermore, FGFR2 activates ERK1/2, which results in inhibition of transcription through the double-strand break repair protein Mre11A [212]. FGFR2 phosphorylation and ERK1/2 activation are reduced in NOD/SCID mouse models xenografted with breast cancer tumours overexpressing FGFR2 followed by treatment with the FGFR2 inhibitor dovitinib (TKI258) [206]. This amplification of FGFR2-dependent signalling might be due to splicing variants of *FGFR2* [213]. For instance, the shorter FGFR2-C3, expressing a short cytoplasmic tail, induces cellular transformation in human mammary epithelial cells because FRS2 is constitutively phosphorylated, even in the absence of any ligand, and robustly activates downstream signalling [213]. Furthermore, the signalling cascades activated by FGF10 downstream of FGFR2b were reported to counteract ER-dependent signalling [156] (figure 4). A potential mechanism for this implies FGFR2-dependent increase in the binding of two transcription factors associated with high-risk breast cancer, NFIB and YBX1, to the ER in the nucleus [159]. Like FGFR1, it is possible that FGFR2 localization in the cytoplasm or nucleus may affect downstream responses (e.g. ER signalling) and clinical parameters [214]. For instance, FGFR2 activation in mammary epithelial cells promotes the activation of the ribosomal s6 kinase 2 (RSK2) downstream from ERK1/2, which results in regulation of FGFR2 intracellular trafficking and increased cell growth and migration [215]. This signalling pathway has been identified in patient material as well, where the lack of FGFR2 and of activated RSK2 significantly correlated with better disease-free survival [215].

FGFR2 signalling promotes HER2 shedding through the metalloprotease ADAM10 and enhances HER2 signalling, HER2-dependent proliferation and tumour progression in mouse xenografts [216] (figure 4). Therefore, FGFR2 may contribute to resistance to HER2 inhibitors. Indeed, FGFR2 inhibitors added to HER2-positive breast cancer cells after failure of treatment with the anti-HER2 drug lapatinib suggest a switch in cell addition to signalling inhibitors [101,217]. Recent data reported that FGFR2 is capable of phosphorylating HER2, leading to resistance both *in vitro* and *in vivo* [137]. Furthermore, the authors showed that FGF5 secreted by cancer-associated fibroblasts (CAF) in the microenvironment might be responsible for the high activation of FGFR2 on the neighbouring epithelial cells [137], confirming the potential signalling switch between HER2 and FGFR2 in breast cancer (figure 4). This idea is in line with novel clinical strategies to treat breast cancer patients with multiple signalling inhibitors, including FGFR1–2 [184,217]. An idea that is worth exploring would be the combination of FGFR2 and EGFR inhibitors, based on the reciprocal regulation of these two RTKs in breast cancer cells *in vitro* [68].

Finally, increased FGFR2 signalling possibly due to the SNP rs2981578 has been studied in stromal fibroblasts responding to FGF10 [155]. On the contrary, FGF7, another specific ligand for FGFR2b (figure 2*a,b*) has been shown to trigger phosphorylation of progesterone receptor at Ser294 and its degradation via the FGFR2–RSK2 signalling axis [151]. In conclusion, FGF7 and FGF10 in the breast cancer microenvironment might regulate FGFR2 signalling-dependent breast cancer cell behaviour through complementary molecular mechanisms. This idea might lead to better therapies if confirmed in patient samples.

### 6.3. FGFR3

*FGFR3* is located on chromosome 4p16.3 and is found mutated in a very low percentage of breast cancer patients, in particular in the extracellular (R248C, S249C) or the transmembrane (G370C, S371C, Y373C, G380R, A391E) protein domains, and more rarely within the kinase domain (K650E, K650N, K650M, K650T, K650Q and N540S) [104]. Gene fusions with *AFF3, AHCYL1, BAIAP2 L, 1SLC45A3, BICC1, PPAPDC1A, TACC1, TACC2, TACC3, NPM1* have also been reported, but not fully characterized [104]. However, there is recent evidence showing that the *FGFR3–TACC3* gene fusion is highly expressed in TNBC cell lines, which results in the autophosphorylation of FGFR3 [218]. Although *FGFR3* gene expression and FGFR3 are rarely found in breast cancer patients, these data suggest a potential oncogenic role for FGFR3. Amplification of the *FGFR3* gene has been found in less than 1% of breast cancer patients [104]. However, FGFR3 expression is associated with ER-positive breast cancers, where it contributes to tumour progression [219]. For instance, FGFR3 expression is increased in tamoxifen-resistant breast tumours and FGFR3 activation in MCF7 cells activates the MAPK, PI3K and PLCγ pathways [220], confirming its putative role in breast cancer development and resistance to endocrine therapy.

### 6.4. FGFR4

The *FGFR4* gene is located on the chromosomal region 5q35.2, and the FGFR4 protein has been found mutated at K535 and E550 in the kinase domain in breast cancer patients, which causes protein autophosphorylation and activation [104]. These mutations are mainly found in metastatic breast cancers [221], suggesting a positive correlation between FGFR4 signalling and metastatic breast cancer. On the same lines, the FGFR4-R388 allele has been associated with tumorigenesis, cell motility and immune evasion [222]. *FGFR4* gene amplification has been found in only 2.3% of breast cancer patients [104], but another study has revealed the presence of FGFR4 mRNA transcript in 30% of patients [134], particularly in metastasis [223]. The discrepancy between gene amplification and the level of transcript or protein can be explained by gene amplification not reflecting protein expression and activity in the case of FGFR4. For instance, high FGFR4 mRNA levels predict failure of treatment with tamoxifen independently from the traditional predictive factors [224]. This would confirm that transcript and protein expression might be better predictors for treatments in breast cancer patients than gene amplification [225].

Few data are available on the association of the *FGFR4* gene or FGFR4 protein with specific breast cancer subtypes. Recently, two studies reported that FGFR4 acts as an important mediator of endocrine resistance and metastasis in invasive lobular carcinoma [221] and luminal A primary breast tumours (HER2-negative) that gives rise to HER2-enriched metastases [112]. Bioinformatics analysis further demonstrated that an FGFR4-induced gene signature predicts site-specific metastasis for lung, liver and brain, but not for bone or lymph nodes [112]. Certainly, FGFR4 cooperates with HER2 to regulate the expression of cyclin D and promote breast cancer cell proliferation [226]. FGFR4 is also a potential mediator of cell survival via activation of PI3 K–AKT signalling [227]. A potential mechanism underlying this effect is FGFR4-mediated regulation of membrane ruffling in response to both FGF1 and 2 [122] (figure 2*a,b*), which might increase AKT signalling. The role of FGFR4 in liver metabolism may offer an alternative explanation to link AKT signalling and survival depending on FGFR4 signalling [222] (figure 4).

A potentially targetable function of FGFR4 is its tumour-delaying effect when metabolism is altered [228]. Although the primary role of FGFR4 in metabolism occurs in hepatocytes, its ablation results in a net inhibitory effect on mammary tumour progression, most likely due to suppressing signals triggered by FGF21 from breast adipocytes [228]. Systemic and microenvironmental metabolic alterations may indeed affect both peripheral and breast adipocytes, thus contributing to the suppression of tumour progression [228], but further studies are needed to confirm this hypothesis. Data showing that FGFR4 is overexpressed in invasive ductal carcinomas and that FGF15/19 signalling mediates the survival of a subset of basal-like breast cancer through FGFR4 and PI3K–AKT signalling confirm the link between metabolism alterations due to FGFR4 levels, survival and an aggressive breast cancer phenotype [227,229]. Furthermore, FGFR4 has been identified as a critical modulator of enhanced glucose metabolism in breast cancer cells, where high levels of FGFR4 not only increase glucose metabolism but also lead to chemoresistance [230]. Finally, the role of FGFR4 in resistance has been recently confirmed also in TNBCs [231], thus expanding the repertoire of breast tumours where FGFR4 signalling plays an important but still understudied role.

## 7. Targeting FGFR signalling in breast cancer

In breast cancer, the development of FGFR inhibitors represents a novel class of drugs. Theoretically, FGFR inhibitors might be positioned in three distinct clinical settings; as a ‘preventive therapy’ prior to a breast cancer diagnosis in high-risk patients, in the adjuvant setting to reduce risk of metastases after surgery for early breast cancer, or in the context of established metastatic breast cancer, to slow the natural history of the disease and thereby improve survival [105]. A GWAS of 1145 post-menopausal patients identified four separate SNPs in the FGFR2 intron associated with breast cancer susceptibility, and meta-analyses of a large cohort of case control studies indicate that such FGFR2-susceptibility SNPs are present across ethnic groups and in different breast cancer sub-groups, predominately hormone receptor-positive disease [232]. Based on FGFR2 interaction with other genetic and environmental factors (reviewed in [233]), FGFR2 may contribute to polygenic risk scoring in breast cancer family history clinics. However, this small contributory role to breast susceptibility is insufficient to support FGFR inhibitors as a candidate for drug prevention in high-risk women.

In the context of drug development of established breast cancer, proof of activity of a candidate protein in the metastatic setting must first be robustly observed. Three important aspects to consider from a clinical development perspective are: (1) Is the incidence of FGFR aberration in metastatic breast cancer clinically meaningful? (i.e. *Do you have the patient population to recruit to a clinical trial in a timely manner and is the patient population of sufficient size to render the drug commercially viable*?); (2) Can FGFR aberrations be promptly and accurately defined before study entry? (i.e. *Are FGFR aberrations easily identifiable in a time-sensitive manner before clinical deterioration from metastatic disease occurs? Can the right drug be offered to the right patient cohort?*); and (3) Is there a partner predictive biomarker to select for those patients who will derive most clinical benefit from FGFR inhibition? (i.e. *Are there clearly defined patient populations to aid commercial development?*). In the case of FGFRs, the presence of activating gene mutations in the FGFR axis, of various forms, has been reported in up to 18% of breast cancers, including *FGFR1* and *FGFR2* amplifications, point mutations in the ligand-binding region and oncogenic fusion proteins [104]. These data would corroborate the idea of FGFR as a clinically meaningful drug target. However, not all the *FGFR* gene aberrations can be easily identified in hospital laboratories by routinely used methods like immunohistochemistry or *in situ* hybridization on tumour biopsies but might require next-generation sequencing (NGS). At present, this level of precision oncology is not routinely available for most patients outside dedicated clinical trials. There is also a significant cost implication when scaled-up to real-world healthcare. The consequence of this potential delay in detecting *FGFR* aberrations is that during the time of the analysis the patient fitness for treatment may deteriorate owing to their burden of metastatic disease, thus making FGFR a difficult drug candidate. In spite of these clinical considerations, of particular interest to drug development is the observation that FGFR dysregulation is associated with the acquisition of endocrine resistance in hormone receptor-positive breast cancer, e.g. in the case of FGFR3 [112]. Multiple reports, including phase 2 clinical trial data, infer a role for the FGFR axis in resistance pathways to well-established metastatic treatment paradigms. For example, poor response to CDK4/6 inhibitors has been observed in patients with coexisting *FGFR1* amplification in hormone receptor-positive breast cancer [184] and dual HER2-targeted therapy in HER2-positive breast cancer [136]. Therefore, the FGFR signalling axis may be positioned as a single agent or in combination, to overcome resistance pathways to established therapies.

In terms of predictive biomarkers of disease response or resistance to FGFR pathway inhibition, FGFR dysregulation has been associated with PI3K, cyclin D1, MYC and p53 mutations in breast cancer [104]. Although it is unclear whether these proteins are independent drivers or exhibit some degree of co-dependence, they may represent potential biomarkers or partner therapeutic opportunities with FGFR inhibitors, together with other potential dysregulated molecular drivers of breast cancer such as the RTK c-MET [234].

### 7.1. Clinical trials of FGFR inhibitors

In the past decade, several approaches have been used to target FGFR using non-selective and selective FGFR inhibitors across several cancer types, including breast cancer. Clinical trial data in breast cancer as a single agent or in combination are summarized in tables 1 and 2, respectively, with a particular focus on small molecule tyrosine kinase inhibitors.

**Table 1.** Clinical trials of single-agent small molecule FGFR inhibitors in breast cancer. PFS, progression-free survival; CBR, clinical benefit rate; ORR, overall response rate; TEAE, treatment-emergent adverse events; DLT, dose-limiting toxicity; MTD, maximum tolerated dose; RP2D, recommended phase 2 dose.

trial identifier	phase	trial design	primary endpoint	current status
AZD4547				
NCT02299999 (SAFIR-02); start date November 2014	2	open label multicentre randomized trial, 1468 participants	PFS (compared with standard maintenance therapy)	active, not recruiting; estimated completion date December 2022
NCT02465060 (NCI-MATCH—breast protocol W); start date June 2015	2	open label multicentre trial, 70 participants	PFS (compared with standard maintenance therapy)	completed
INCBO54828 (pemigatinib)				
NCT03822117 (FIGHT-207); start date January 2019	1	open label multicentre trial in patients with activating FGFR mutations or translocations, 170 participants, three cohorts— Cohort A: solid cancers with FGFR1–3 in-frame fusions; any FGFR2 rearrangement; FGFR1/3 rearrangement with known partner. Cohort B: solid cancer with activating mutations (excluding kinase domain) in FGFR1–3. Cohort C: solid cancers with FGFR1–3 known activating mutations in kinase domain; FGFR1–3 putatively activating mutations; other FGFR1/3 rearrangements	ORR	active, and recruiting; estimated completion date March 2022
BAY1163877 (rogaratinib)				
NCT04125693; start date October 2019	2	open label	TEAE	completed
RLY-4008				
NCT04526106; start date August 2020	1	open label first in human clinical trial in patients with solid cancers and activating FGFR2 mutation, FGFR2 fusion or FGFR2 amplification	MTD and RP2D	active and recruiting; estimated completion date October 2024
TAS-120 (futibatinib)				
NCT02052778; start date February 2014	1	open label dose escalation and expansion study and phase 2, 386 patients	ORR	active, not recruiting; estimated completion date June 2022

**Table 2.** Clinical trials of FGFR inhibitors and potential combination therapy in breast cancer. PFS, progression-free survival; CBR, clinical benefit rate; ORR, overall response rate; TEAE, treatment-emergent adverse events; MTD, maximum tolerated dose; DLT, dose-limiting toxicity; RP2D, recommended phase 2 dose.

trial identifier	phase	trial design	combination therapy	primary endpoints	current status
dovitinib					
NCT01528345; start date February 2012	2	dovitinib	fulvestrant	PFS	early study termination; results available
debio-1347					
NCT03344536; start date November 2017	1b/2	open label, non-randomized FGFR-amplified ER+ metastatic breast cancer	fulvestrant	DLT (phase 1), ORR (phase 2)	active, not recruiting; estimated completion date August 2021
INCB054828 (pemigatinib)					
NCT02393248 (FIGHT 101); start date March 2019	Phase 1/2	open label, dose-escalation, safety and tolerability study, 201 participants	gemcitabine + cisplatin + pemigatinib, pembrolizumab + pemigatinib, docetaxel + pemigatinib, trastuzumab + pemigatinib, INCMGA00012 + pemigatinib	MTD, pharmacodynamics as monotherapy and in combination	active, not recruiting; estimated completion date December 2021
AZD4547					
NCT01202591 (GLOW); start date September 2010	2	open label randomized trial in ER+ breast cancer patients with FGFR1 polysomy (FISH4/5) or gene amplification (FISH 6)	fulvestrant, exemestane	safety and tolerability	completed; results available
NCT01791985 (RADICAL); start date February 2013	1b/2	open label AZD4547 in combination with either anastrozole or letrozole in ER+ breast cancer patients progressing on these aromatase inhibitors	anastrozole, letrozole	safety and tolerability	completed; results available
JNJ-42756493 (erdafitinib)					
NCT03238196; start date August 2017	1b	open label, non-randomized in 35 patients with ER+/HER2−/FGFR− amplified MBC	fulvestrant, palbociclib	safety and tolerability of combination therapy	active, not recruiting; estimated completion date December 2022
TAS-120 (futibatinib)					
NCT04024436 (FOENIX); start date July 2019	2	open label non-randomized cohort design 168 patients: Cohort 2— TNBC measurable disease, FGFR2 amplification; Cohort 3—HR+ HER2− or TNBC non-measurable disease, FGFR2 amplification; cohort 4—HR+ HER2− measurable disease, FGFR1 amplification	fulvestrant	ORR, CBR and PFS	currently recruiting; estimated completion date June 2023
BGJ398 (infigratinib) (FGFR1–3-selective)					
NCT04504331; start date August 2020	1	open label first in-combination clinical trial, 10 participants, HR+, HER2−, FGFR altered advanced breast cancer	tamoxifen or fulvestrant+palbociclib	DLT	currently recruiting; estimated completion date October 2023

#### 7.1.1. Single-agent clinical trials

Proof-of-concept studies of FGFR pathway utility as a clinical target in solid tumours were initially performed using non-selective FGFR inhibitors in phase 1 studies. Examples of such non-selective agents include dovitinib (TKI258), which has activity against FGFR1–3, VEGFR1–3 and PDGFR, lucitanib, which has activity against FGFR1–2, VEGFR1 and colony stimulating factor receptor, and lenvatinib, with activity against FGFR1–4, KIT, RET and PDGFR beta [100,101,119,179,235,236]. Of course, multiple receptor targets can lead to multiple mechanisms of drug toxicity and therefore a move towards more selective FGFR drug development has been prioritized (table 1).

With increasing selectivity (and indeed sub-selectivity) for FGFRs, FGFR inhibitors have been assessed for dose, safety and tolerability within larger platform or basket trials to facilitate patient selection by NGS of tumour tissue or circulating tumour DNA. In NCI-MATCH [237], a platform trial in which drug selection was dictated by molecular profile rather than tumour histology, patients with cancers with targetable mutations in an FGFR pathway were treated with AZD4547, an orally bioavailable tyrosine kinase inhibitor, selective for FGFR1–3, until disease progression. Of the 70 patients assigned to AZD4547, 33% had metastatic breast cancer. The predominant FGFR aberration recorded was FGFR1 amplification or activating mutations in FGFR2 or 3. Response rates across the trial cohort were low (8%), consistent with the heavily pre-treated study population, and differed by mechanism of FGFR dysregulation. For example, patients with FGFR fusions showed the highest response rate of 22% to AZD4547 (90% CI 4.1–55%), with six-month progression-free survival (PFS) standing at 56%. In non-responders, there was a high prevalence of concurrent dysregulation of the PI3K/AKT/mTOR pathway. Tolerability of the drug was good, with most side effects concerning skin or mucus membranes and low grade in nature. The tumour-specific SAFIR-02 (NCT02299999) clinical trial is an open label phase 2 platform study that assigns drug treatment to metastatic breast cancer patients based on genomic profiling, compared with standard maintenance chemotherapy and/or immunotherapy. This study has recently closed to recruitment and results are anticipated soon. The study design includes AZD4547 for treatment of FGFR-dysregulated metastatic breast cancers, as assessed by high-throughput sequencing of frozen tumour tissue or circulating tumour DNA (ctDNA). In the FIGHT-207 multicentre phase 1 basket trial (NCT03822117), patients with solid cancers were assigned to the treatment cohorts dependent on FGFR dysregulation type: Cohort A included FGFR–13 in-frame fusions, FGFR2 rearrangements or FGFR1/3 rearrangements with a specific partner; C–ohort B included non-kinase domain activating FGFR13 mutations; and Cohort C, kinase domain activating mutations. All patient cohorts were given oral p–emigatinib, an inhibitor of FGFR13 signalling transduction, which has recently been granted accelerated FDA approval for FGFR2-amplified cholangiocarcinoma [238]. The FIGHT-207 study has recently closed to recruitment and results are awaited. A further tumour agnostic basket trial, denoted FUZE (NCT03834220) is currently assessing objective response rate to Debio-1347 in biliary, urothelial and solid cancers (including breast cancers) with FGFR1–3 fusions or rearrangements, based on encouraging phase 1 data. FGFR fusion screening is performed using whole-transcriptome sequencing, with a turnaround time of 14 days, a timeframe that is consistent with real-world clinical application. Planned recruitment is for 125 patients with interim futility/homogeneity analysis after 27 patients are on the study.

Rogaritinib (BAY1163877) is a potent FGFR1–4 small molecule inhibitor that has recently completed dose-finding and safety assessment in the phase 1 trial setting [239] with activity predominately in urothelial, non-small lung cancers and cholangiocarcinoma (time to progression 60–100 days), where FGFR overexpression was detected by mRNA expression. In the unselected solid tumour cohort (including breast cancers), median time on drug before progression was 47 days. Grade 3 toxicity was seen in fewer than 9% of patients (fatigue, anaemia, urinary tract infection). However, grade 1 or 2 hyperphosphataemia, anorexia or diarrhoea, which may still have meaningful detriment to quality of life in the metastatic setting, was seen in more than 33.3% of patients on the study [239]. Using a more traditional phase 1 clinical trial design, futibatinib, an orally bioavailable, irreversible inhibitor of FGFR1–4 demonstrated an overall response rate of 13% across several solid tumour types, including breast cancer [240]. Futibatinib is non-ATP-dependent and binds irreversibly to a cysteine loop in the receptor kinase region. Of the 170 patients assigned to this study, 50% had FGFR fusions or rearrangements, 30% had FGFR-activating mutations and 14% FGFR amplifications. Of the patients, 6.5% had a diagnosis of metastatic breast cancer with the majority having received between two and four prior lines of therapy. Responses to futibatinib were observed across the full spectrum of FGFR aberrations but were more commonly found in cholangiocarcinoma and primary central nervous system (CNS) cancers. Within the breast cohort, 3/11 patients showed some evidence of tumour shrinkage, with one FGFR2-amplified TNBC patient showing a prolonged partial response to futibatinib treatment, leading to further characterization of this drug in an ongoing phase 2 study (FOENIX, NCT04024436) in FGFR1- and FGFR2-amplified triple negative or hormone receptor-positive breast cancer.

In conclusion, there are several promising clinical trials based on single agents for which results are awaited that may change the way we treat patients with dysregulated FGFRs.

#### 7.1.2. Combination-agent clinical trials

There has been increasing interest in developing novel partners to endocrine therapy, to overcome the inevitable progression to acquired endocrine resistance, after first-line single-agent aromatase inhibitors or aromatase inhibitors in combination with CDK4/6 inhibition [184] (table 2). In phase 2 data, fulvestrant (a selective oestrogen degrader—SERD (NCT03455270)) was combined with non-selective FGFR1–3 inhibitor dovitinib in hormone receptor-positive and *FGFR1*-amplified post-menopausal patients who had relapsed on or shortly after endocrine therapy. The study was slow to recruit owing to low identification rate of *FGFR1* amplification in the study cohort, and terminated early, with a lower number than expected survival events in the dovitinib arm. In the non-selected study population, there was no survival benefit on the addition of dovitinib to fulvestrant (median progression-free survival (PFS) 5.5 months in both placebo and dovitnib group). In the *FGFR1*-amplified cohort, there was a modest survival benefit (10.9 months dovitinib arm versus 5.5 months placebo), which met pre-defined superiority criteria [241]. A comparable study using the non-selective FGFR1–3 inhibitor lucatinib in combination with fulvestrant in post-menopausal women after disease relapse on endocrine therapy was again terminated early owing to slow recruitment (18 patients in total received drug) [242]. In this study, a partial response was observed in 3/18 patients. However, 78% of patients developed grade 3 hypertension, necessitating dose reduction, which is a likely side- effect from co-inhibition of the VEGF axis [242].

Turning towards selective FGFR tyrosine kinase inhibition, two trials using AZD4547 have addressed the question of acquired endocrine resistance in hormone receptor-positive metastatic breast cancer. In RADICAL (NCT01791985), 58 post-menopausal women, who had developed progressive disease with aromatase inhibitors letrozole or anastrozole, were given AZD4547 alongside their pre-existing endocrine therapy, with an aim to re-sensitize to endocrine treatment. At 28 weeks, there were two partial responses to combination therapy and 11 patients had stable disease. Combination treatment was tolerable, with frequently reported side effects including hyperphosphataemia, hair loss and nausea . In the GLOW clinical trial (NCT01202591), post-menopausal metastatic breast cancer patients, who had progressed on first-line endocrine therapy, were offered AZD4547, in combination with exemestane (an irreversible steroidal aromatase inhibitor) or fulvestrant, using different dosing strategies. On this occasion, patients were pre-screened for FGFR1 polysomy (FISH4/5) or gene amplification (FISH6), using *in situ* hybridization assessment in local laboratories before study entry. However, the low number of eligible patients led to slow recruitment and early termination of the study after 40 patients (initial planned cohort 127 patients) based on commercial decision-making. Adverse events listed included anaemia, alopecia, hyperphosphataemia and anorexia. Monitoring for eye disorders was undertaken based on pre-clinical data. Dry eyes or excess lacrimation was the most common listed event and mild in nature, in this limited study. AZD4547 was subsequently licensed by Astra Zeneca to Abbisko in 2019 for further research and development.

By contrast, FOENIX (NCT04024436), a phase 2 clinical trial of futibatinib in hormone receptor-positive or triple negative metastatic breast cancer is recruiting well, with a target accrual of 168 patients, across diverse geographical regions. Cohort 4 of this study assesses the utility of combination therapy with fulvestrant in *FGFR1*-amplified hormone receptor-positive cancer, with results anticipated to be available in 2023/2024.

Two FGFR inhibitors are currently undergoing assessment for efficacy in combination with CDK inhibitors and endocrine therapy in hormone receptor-positive breast cancer in small phase 1 trials. Erdafinitib (JNJ-42756493) is a small molecule pan-FGFR pathway inhibitor that has recently gained FDA approval in urothelial cancers. The study schedule in FGFR-amplified breast cancer (NCT03238196) is to recruit 35 patients to assess efficacy and safety in combination with fulvestrant and palbociclib (after prior disease progression on aromatase inhibition). Secondary endpoints include predictive biomarker evaluation to aid patient selection, putatively based on amplification status of FGFR1–4, CDK4 or 6, and cyclin D1/2 and also mutation status of RB1 and ESR. Infigratinib (BGJ-398), a selective FGFR1–3 inhibitor, is in early stage of evaluation for efficacy and tolerability in metastatic breast cancer. Study design includes expansion to combination therapy with tamoxifen or fulvestrant/palbociclib as potential therapeutic co-targets (NCT01928459).

Using another therapeutic approach, FIGHT 101 (NCT02393248) is currently evaluating the addition of pemigatinib to a number of well-characterized cytotoxic chemotherapeutics (gemcitabine, cisplatin, docetaxel), HER2-targeted therapy (trastuzumab) and immunotherapy (pembrolizumab) across solid tumours, including breast cancer. The interaction between inhibition of the FGFR pathway with HER2-targeted therapy and immunotherapy, is awaited with some interest.

### 7.2. FGFR inhibitors: tolerability and toxicity

Apart from efficacy, a key consideration in drug development of molecular-targeted therapies (MTTs) is patient tolerability and drug toxicity. Drug toxicity can occur as a result of ‘on-target, off-cancer’ effects or idiosyncratic ‘off-target’ effects of the drug. Drug tolerability, the ability of a patient to maintain quality of life while on an effective anti-cancer therapy, is a crucial issue, particularly in the context of declining fitness (performance status) with metastatic disease. Germline FGFR mutations are associated with defects in bone development and phosphate metabolism. This has led to increased monitoring of bone metabolism and associated ectopic mineralization with FGFR inhibitors in pre-clinical and clinical studies. However, to date most phase 1 and phase 2 clinical trials have reported side effects of FGFR inhibition to be mild or moderate, with more common patient reported symptoms– including dry skin, dry mouth, nausea and diarrhoea. Ophthalmological assessment is built into patient pathways, to ensure early recognition of rare corneal or retinal effects of FGFR inhibitors. In terms of laboratory results, mild–moderate elevation of plasma phosphate (hyperphosphataemia) and liver enzymes has been commonly reported, well mitigated by a robust clinical care plan. Increased specificity, avoiding VEGFR co-inhibition, has increased tolerability and reduced adverse events. Similarly, avoiding FGFR4 co-inhibition has reduced unpleasant gastrointestinal symptoms such as diarrhoea. In pre-clinical studies, FGFR inhibition with small molecule inhibitors has been associated with embryonic lethality in rodents and therefore effective contraception is mandated with FGFR inhibitors for patients of child-bearing age and their partners. Further safety data regarding rarer side effects of FGFR small molecule inhibitors will be forthcoming with reporting of large-scale phase 2 trials [3].

## 8. Conclusion and perspectives

In the last few years, large-scale studies based on new technologies have revealed how drivers of breast cancer, including FGFRs, their ligands and signalling partners, are dysregulated [243]. Here, we have discussed the presence of various genomic alterations, the lack of signalling regulation, and impaired paracrine or autocrine activation in the context of FGFR signalling and in the context of ongoing clinical trials. Our increased understanding of these mechanisms has implications for selecting and improving anti-breast cancer therapies where FGFRs are dysregulated. However, despite the amount of available information there are still several aspects of FGFR signalling in breast cancer that are worth further investigation, from both a cellular and a clinical perspective.

### 8.1. What is missing from a signalling perspective?

Little is known about the specific role of the splicing variants of *FGFR* genes in breast cancer initiation, development or therapeutic targeting [244]. Given the variety of phenotypes of mice knocked-out for *Fgfr* isoforms [4], the differential expression and role of the ‘b’ and ‘c’ variants in tissue [61], and the possibility of trans-phosphorylation between different FGFRs [245], it is worth speculating that the landscape of dysregulated FGFR signalling is much more complex than the one presented here (figure 4). Several recent publications have correlated members of all the FGF subfamilies, and not only the well-characterized FGF7 subfamily [2], to diverse aspects of breast cancer development. This information may be used to uncover which FGFR isoforms are most likely to be activated in different breast cancer subtypes, in addition to available data on the status of the gene of each FGFR in that subtype [3]. For instance, there are indications that FGFR3 plays a role in ER-positive tumours despite its low expression in clinical samples [219]. The FGFR3 ligand FGF8 has been associated with increased proliferation and cell cycle progression in ER-positive tumours [164,165]. It would be therefore worth investigating the correlation between FGF8 and the FGFR3c or the FGFR3b isoform [246,247], besides focusing on the autocrine role of FGF8 through FGFR1 and FGFR4 in epithelial cells. It would be also worth investigating the switch between epithelial ‘b’ and mesenchymal ‘c’ isoforms, for instance in the case of the *FGFR2* gene, as this switch contributes to changes in ligand binding and intracellular signalling, as also shown in prostate cancer [248,249]. To decipher the contribution of each FGF/FGFR pair to breast cancer progression there is an unmet need for specific biochemical tools to detect FGFR isoforms and for novel methods—including advanced single- cell mRNA sequencing, quantitative phosphoproteomics and proteogenomic and high-resolution imaging—to be performed in breast cancer models grown in three-dimensional culture or in patient-derived organoids [225]. A complete understanding of the role of FGFR signalling in breast cancer biology and of how FGFR signalling architecture adapts during breast cancer progression [250] or treatment with canonical strategies will then pave the way for discovering or improving novel strategies to benefit breast cancer patients. For instance, the use either of certain FGFs as therapeutics or of more specific ligand-trap molecules and isoform-specific FGFR inhibitors still needs further optimization [2,133,251–254].

### 8.2. What is missing from a clinical perspective?

To date, FGFR inhibitors have shown promising clinical activity in tissue agnostic, biomarker-driven ‘basket’ clinical trials and tissue-specific ‘umbrella’ trials, including exciting response rates in previously treatment-recalcitrant cancers such as cholangiocarcinoma [238,255,256]. In breast cancer, the clinical incidence of *FGFR1* amplification is 10% and that of *FGFR2* amplification is approximately 2%, and these amplifications are seen across all clinical subtypes. Point mutations in FGFR receptors are less frequent. On a practical level, this necessitates identification of a potentially treatment-sensitive patient cohort from the larger breast cancer population by screening for FGFR amplification/mutation. Screening large numbers of patients is arduous, time-consuming and currently non-routine outside a clinical trial or in early disease. This therefore represents a potential barrier to uptake of FGFR inhibitors. Approval and adoption of FGFR inhibitors in breast cancer will stand or fall on the results of large-scale phase 2/3 trials of the use of FGFR small molecule inhibitors as monotherapy or combination therapy in breast cancer (tables 1 and 2). To date, toxicity has been predictable and manageable within clinical care plans for patients with a reasonable fitness level. An area of significant interest is the role of FGFR inhibitors in the endocrine-resistant breast cancer population, as discussed above.

## Data accessibility

This article has no additional data.
